# Prospective Study of Endogenous Estrogen History and Cognitive Decline in the Nurses' Health Study

**DOI:** 10.1002/alz.088911

**Published:** 2025-01-03

**Authors:** Namuunaa Juramt, Jae H. Kang, Dong D. Wang, Chirag M. Vyas, Cheng Peng, Sharon Curhan, Erica T. Warner, Oana A. Zeleznik, Bernard A. Rosner, Molin Wang, Meir J. Stampfer, Olivia I. Okereke, A Heather Eliassen

**Affiliations:** ^1^ Channing Division of Network Medicine, Brigham and Women’s Hospital and Harvard Medical School, Boston, MA USA; ^2^ Channing Division of Network Medicine, Brigham and Women’s Hospital, Harvard Medical School, Boston, MA USA; ^3^ Harvard T. H. Chan School of Public Health, Boston, MA USA; ^4^ Department of Psychiatry, Massachusetts General Hospital, Harvard Medical School, Boston, MA USA; ^5^ Massachusetts General Hospital and Harvard Medical School, Boston, MA USA

## Abstract

**Background:**

Endogenous estrogen history across the life course may be associated with better cognitive maintenance. Few large longitudinal studies have evaluated this prospectively, and results have been inconsistent. We assessed the association of reproductive span, an indicator of endogenous estrogen history, with cognitive change in older women.

**Method:**

We followed 13,419 Nurses’ Health Study participants who were free of stroke, were ≥70 years old as of 1995‐2001, reported natural menopause or bilateral oophorectomy and had information on ages at menarche and menopause. Reproductive span was defined as age at menopause minus age at menarche. Four telephone‐based cognitive assessments were administered (at 1.5‐2 year intervals). The primary outcome was the global composite score, averaging z‐scores of 6 tests measuring general cognition (the Telephone Interview of Cognitive Status, TICS), verbal memory, category fluency, and attention. Linear mixed‐effects models, adjusted for age, education, depression, menopausal hormone therapy (MHT) within 10 years of menopause, surgical menopause, and other lifestyle and health variables, were used to estimate the differences in annual rate of change over time by quintiles (Qs) of reproductive span. We examined interactions with menopause type, MHT duration and apolipoprotein E (*APOE*) e4 allele (among 5,434 women).

**Result:**

The mean baseline age was 74.3 years, the mean follow‐up was 6.6 years, and the mean reproductive span was 36.4 years (range = 7‐46 years). Longer reproductive span was significantly associated with more favorable cognitive change: compared with women with the shortest reproductive span (Q1: ≤33 years), women with the longest reproductive span (Q5: ≥41 years) demonstrated better maintenance in global cognition (difference in annual rate of change_Q5vs.Q1_ = 0.007; 95% CI: 0.00001, 0.01; p‐trend = 0.02); this difference was equivalent to that observed in women 1.4 years apart in age. We observed similar trends with TICS and verbal memory (p‐trends≤0.04), but not category fluency (longer reproductive span was associated with better baseline performance, p≤0.01; but not differences in rates of decline) and attention (no associations found). We observed no interactions with surgical menopause, MHT, or *APOE* e4.

**Conclusion:**

Longer reproductive span, an indicator of greater endogenous estrogen history, was significantly associated with more favorable cognitive maintenance in older women.

